# The Sewing of Wounds

**Published:** 1898-05

**Authors:** 


The Sewing of Wounds.—Prof. Fröliner sewed up a wound in the hindleg of a horse, which was about 58 cm., long and 20 cm. deep, and which extended from the thigh to the outer angle of the haunch-bone. The wound was comparatively fresh. The treatment was: disinfecting irrigations for a quarter of an hour; sewing the wound with sterilized silk; draining and keeping open the lower corner; on the fourth day a rise of 1° in temperature and incipient necrosis at one point in the thread. He then removed the silk, used antiseptic irrigations and a subcutaneous injection of spirits of camphor. In spite of this the horse died of septicæmia the following night. The postmortem examination showed necrosis at the edges of the wound, and the symptoms of septicæmia. This would probably not have occurred if the wound had received open treatment. Since that time the sewing of large and not entirely fresh wounds has been discarded, and the slower but less dangerous open treatment preferred.—*Idem.*

